# Materials informatics for developing new restorative dental materials: a narrative review

**DOI:** 10.3389/fdmed.2023.1123976

**Published:** 2023-01-26

**Authors:** Satoshi Yamaguchi, Hefei Li, Satoshi Imazato

**Affiliations:** Department of Biomaterials Science, Osaka University Graduate School of Dentistry, Suita, Osaka, Japan

**Keywords:** artificial intelligence, deep learning/machine learning, materials informatics, CAD-CAM, digital dentistry, restorative dental materials

## Abstract

Materials informatics involves the application of computational methodologies to process and interpret scientific and engineering data concerning materials. Although this concept has been well established in the fields of biology, drug discovery, and classic materials research, its application in the field of dental materials is still in its infancy. This narrative review comprehensively summarizes the advantages, limitations, and future perspectives of materials informatics from 2003 to 2022 for exploring the optimum compositions in developing new materials using artificial intelligence. The findings indicate that materials informatics, which is a recognized and established concept in the materials science field, will accelerate the process of restorative materials development and contribute to producing new insights into dental materials research.

## Introduction

Materials informatics (MI) is a field of research in materials science, and its significance has increased steadily in the discovery of new materials such as alloys (1–3), polymers (4, 5), and ceramics (6). Some of the materials have been successfully synthesized according to the discovery (7–9). The term “materials informatics” was first used in 2003 by Rodgers JR (10) and defined as “the application of computational methodologies to processing and interpreting scientific and engineering data concerning materials.” The publications related to this field have dramatically increased since 2015 (11), a few years after the statement of the Materials Genome Initiative in 2011 (12). The success of deep learning (13) and big data (14, 15) has also triggered an acceleration in MI studies.

**Figure 1:**
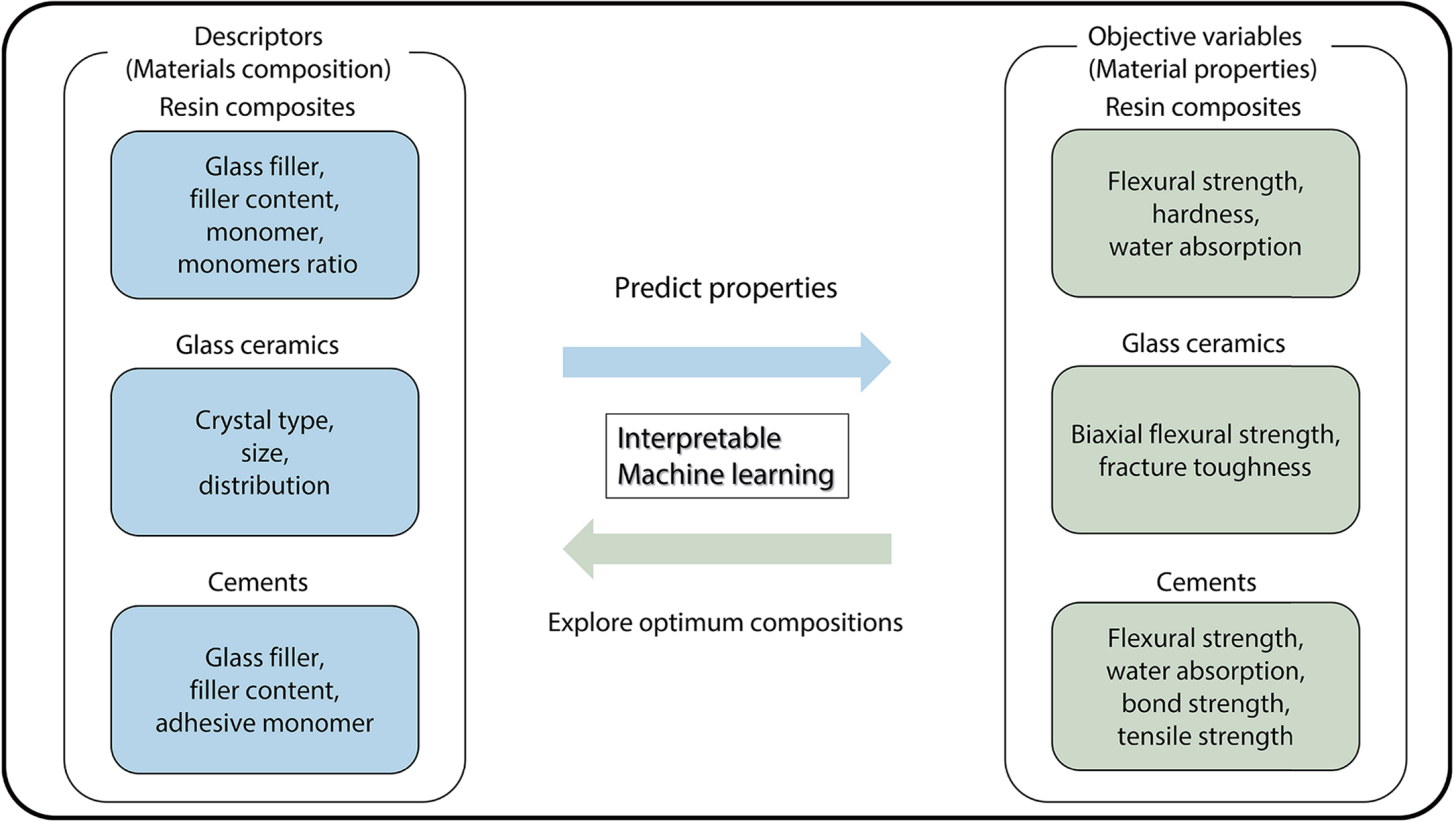
Schematic illustration of the materials informatics approach.

Machine learning, which is a broader concept of deep learning (16), is a data analytics technique that employs artificial intelligence (AI) to explore the regulations underlying datasets by defining clear relationships between input and output datasets from *in vitro* experiments. It has recently become a major tool in MI and has been used for the prediction of material properties as a solution to a direct problem from unknown features (compositions, experimental conditions, etc.) (17) that cannot be used for the development of a regression model. Compared to the conventional method based on density functional theory requiring high-performance computer clusters, the machine learning models can be developed with minimum computer resources (18). In MI, solving an inverse problem to derive features for achieving desirable material properties is particularly important for discovering new materials (Figure 1).

In the dental field, Li et al. were the first to apply the MI approach to predict the flexural strength of computer-aided design/computer-aided manufacturing (CAD/CAM) resin composites, and they successfully explored the optimum compositions to achieve desirable flexural strength (19). Thus, the MI approach promises to make dental material research more efficient than the conventional trial-and-error approach (20).

This narrative review comprehensively summarizes the advantages, limitations, and future perspectives of MI from 2003 to 2022, particularly focusing on the methodology to explore the optimum compositions and thereby achieve the desired properties of dental materials using machine learning approaches.

## For restorative materials and their important properties

### Resin composites

Resin composites as indirect restorative materials consist of a glass filler, monomers, and a silane coupling agent (21). Flexural strength is the most typical mechanical property for evaluating the fracture and deformation resistance of resin composites (22) and can be measured by a three-point bending test according to ISO 4049:2019 (23). Filler press and monomer infiltration have been established to fabricate computer-aided design/computer-aided manufacturing (CAD/CAM) resin composites (24). The flexural strength of such CAD/CAM resin composites is significantly better than that of resin composites for filling and is acceptable for posterior tooth restorations (25). However, CAD/CAM resins still have lower flexural strength than glass-ceramic materials (26) because of the degradation of the silane coupling agent (27).

### Glass ceramics

Ceramics are widely used as indirect restorative materials owing to their high biocompatibility and pleasing aesthetics (28). Lithium disilicate glass ceramics are the top material choice for anterior tooth restorations as a single-unit crown (29). The pre-crystallized state contains metasilicate and lithium disilicate nuclei, which are recrystallized by heat treatment. After heat treatment, the flexural strength increases dramatically (28). Recently developed lithium disilicate glass ceramics do not require any firing after milling (30) and are expected to reduce chair time. In this regard, the MI approach will fit the recent lithium disilicate glass ceramics, that is, no phase change. In addition to the three-point bending test, the biaxial flexural strength test (31) is commonly used to evaluate the flexural properties of lithium disilicate glass ceramics (32).

### Resin/glass ionomer cements

The long-term clinical success of dental restorations depends, in part, on the use of luting cements and cementation procedures. The main task for luting cements is to provide an impervious seal between the abutment and the restoration (33). Resin composite cement and glass ionomer cement (GIC) are widely used types of dental adhesives (34). The conventional GIC is made of calcium fluoro-aluminosilicate glass powder combined with water-soluble polycarboxylic acid. Resin composite cements can be divided into adhesive and self-adhesive resin cements (35). The former has a composition similar to that of restorative resin composites, with a lower filler concentration to ensure a thin film thickness and an acceptable working time (36). The latter allows tooth restoration adherence without the use of separate adhesives and etchants. The major constituents of self-adhesive resin cement include functional acidic monomers, conventional dimethacrylate monomers, fillers, and activator-initiator systems (35). Mechanical strength and handling properties are important properties to consider when using different luting cements. For flexural strength testing, the testing method for resin composites and cements specified in ISO 4049:2019 is usually adopted. However, because the luting cement applied for fixed prostheses is formed as a thin layer, Kawashima et al. proposed an evaluation method to assess the mechanical strength (flexural, tensile, and shear strength) of film-formed self-adhesive resins reflecting cement thickness (37). A consistency evaluation method to determine whether a resin cement to be tested has appropriate flowability for the setting of prosthetic appliances was also proposed by the same author (38).

## Materials informatics

### Data preparation

Descriptors (*x*) such as material compositions (e.g., filler, monomers, and silane coupling agent) and synthesis conditions (e.g., pressure and temperature) for the material properties (*y*) (e.g., flexural strength) are defined according to human knowledge from *in vitro* experiments (e.g., three-point bending test). Descriptors are commonly normalized (from 0 to 1) and standardized (mean = 0, standard deviation = 1) to avoid non-convergence. To develop a good generalization model, descriptors should be appropriately selected from the experimental data.

### Regression model development

**Figure 2:**
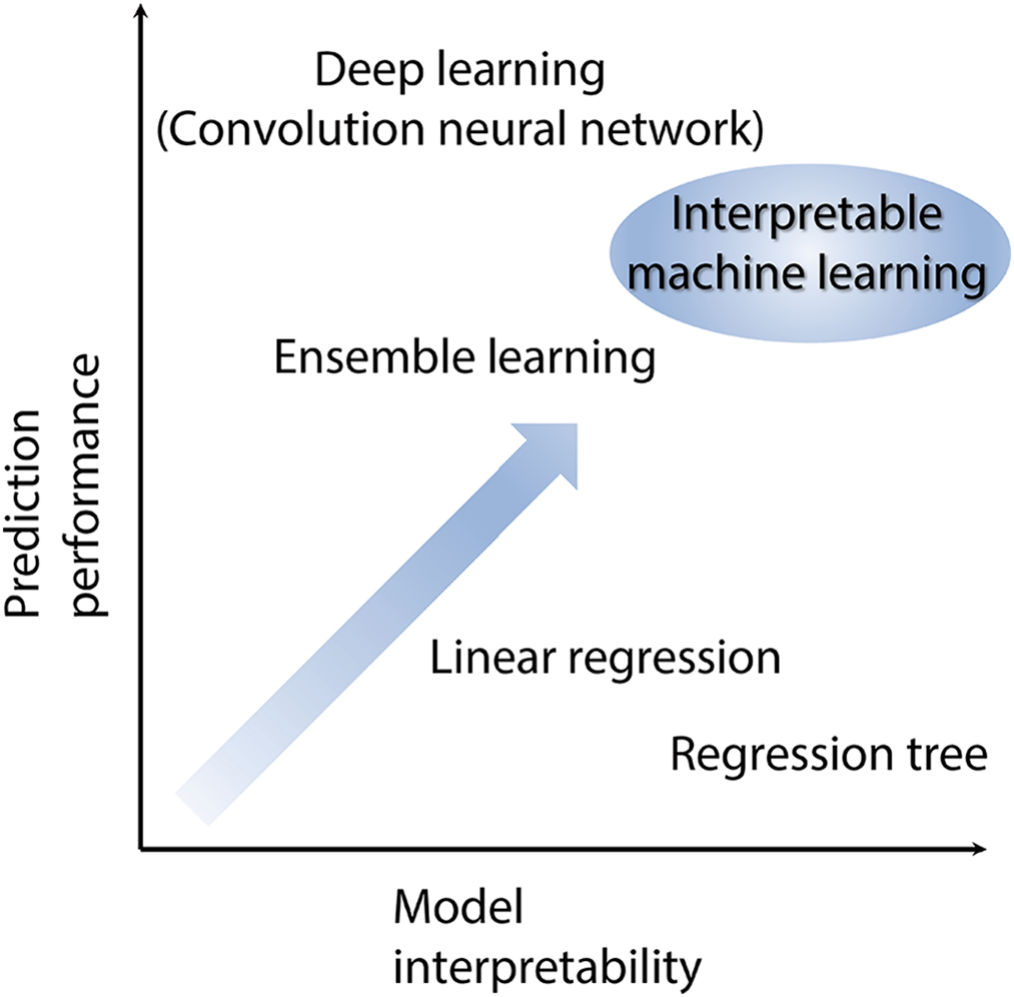
Relationship between prediction performance and model interpretability.

Machine learning can be categorized into supervised and unsupervised learning. Supervised learning is represented by the following equation:

(1) $$y=f\left(x_{1},x_{2},x_{3},\cdots ,\;x_{M}\right),$$

where *x* is the descriptor in MI and *y* is the objective variable. When the objective variable is represented by consecutive numbers, to solve this equation, the relationship between *x* and *y* is called regression. A fitted line or curve can be drawn using a machine learning algorithm. This fitted line or curve is called the regression model. In cases involving many descriptors, fitting on plots is difficult, and machine learning algorithms such as neural networks, support vector machines, and random forests are required. In the MI approach, interpretable machine learning is useful for obtaining new ideas to determine the optimum descriptors for new materials. The relationship between prediction performance and model interpretability is a tradeoff, as shown in Figure 2.

### Model evaluation

The most appropriate relationship between descriptors (*x*) and objective variables (*y*) can be determined by identifying the most appropriate hyperparameters for the selected algorithms to represent the relationship. During this process, the *in vitro* dataset was often divided into training and test data to avoid overfitting, wherein the identified relationship fit the training data well, but was unable to fit unseen data in the testing set (39). The training dataset was randomly split into two groups: 80% or 70% (depending on how large the dataset is) of the data was used for training the model, and the remaining 20% or 30% was used for testing. For the training dataset, to further avoid overfitting, the *k*-fold cross-validation method was used, in which the model fits the training data *k* times. For each iteration, the training data were split into *k* subsets; *k−1* subsets were used to train the model, and the *k*^th^ subset was used as the test data. The hyperparameters that exhibited the best performance during the cross-validation process were selected for the machine learning models. The coefficient of determination (*R^2^* value), root mean square error (*RMSE*), and mean absolute error (*MAE*) were used to assess the regression accuracy of the trained machine learning models. These metrics are expressed as follows:

(2) $$R^{2}=1-\frac{\sum_{i=1}^{m}{\left({\hat{y}}^{\left(i\right)}-y^{\left(i\right)}\right)}^{2}}{\sum_{i=1}^{m}{\left(\bar{y}-y^{\left(i\right)}\right)}^{2}}\;\;$$

(3) $$RMSE=\frac{1}{m}\overset{m}{\underset{i=1}{\sum }}{\left(y^{\left(i\right)}-{\hat{y}}^{\left(i\right)}\right)}^{2}\;\;$$

(4) $$MAE=\frac{1}{m}\overset{m}{\underset{i=1}{\sum }}\left|y^{\left(i\right)}-{\hat{y}}^{\left(i\right)}\right|\;\;$$

where $y^{\left(i\right)}$ was the material properties obtained from *in vitro* experiments, ${\hat{y}}^{\left(i\right)}$ was the predicted material properties from the trained machine learning algorithms, and m was the number of test samples. $R^{2}$ values close to 1 indicated good predictability of the model, while the other two indices close to zero indicated good predictability.

### Optimum descriptor search

If a good regression model is successfully developed, the optimum descriptors that achieve desirable material properties can be inversely searched. The simplest method is an exhaustive search using a linear regression model. From all combinations of descriptors, the material properties are predicted according to the regression model, and the material property with the best performance can be selected. However, in searches involving many descriptors, the prediction process will be time-consuming. Bayesian optimization (BO) can overcome this issue by developing non-linear regression models (also called “surrogate model”) and acquisition functions. The commonly used probability distribution model is Gaussian process regression, which estimates the mean and variance of the training data as a posterior distribution. However, the dimensions of the descriptors could be large, resulting in a large reaction space of up to tens of thousands of possible compositions that cannot be all conducted *in vitro* to update the posterior distribution. Therefore, after training the surrogate model, an acquisition function was used to select the next trial experiment from the reaction space. There are two typical strategies for acquisition functions: exploration and exploitation. Exploitation tends to select the next experiment around the neighborhood of the current best observed value, while exploration tends to select the next point with the greatest predictive uncertainty and tends to investigate the entire reaction space thoroughly (40). Commonly used acquisition functions such as expected improvement (EI) aim to balance these two strategies. Shields et al*.* used the BO method to optimize the yield of two reactions in the pharmaceutical field and successfully found unconventional compositions and configurations that were not commonly selected by human experts, and improved the yield within only 40 experiments (40). Overall, an exhaustive search could be considered when dealing with a small reaction space; however, in searches involving a large reaction space with varied compositions, concentrations, temperature, and pressure, such as the development of new dental materials, all failure datasets accumulated during the developing process could be used as training data, and the BO method could be considered to accelerate the procedure for finding the new formulations.

## Limitations and future perspectives

The MI approach has opened the door to accelerating the discovery and design of new dental materials. However, the synthesis of such new dental materials is still difficult owing to the complexity of the manufacturing process. With further advancements in dental materials research, process informatics (PI) (41, 42), which is the methodology for synthesizing actual materials on the basis of the MI approach, will receive more attention. Building a sustainable open database to accumulate information regarding various manufacturing processes, regardless of success or failure, is important to achieve PI. The autonomation of the manufacturing process using machine learning (43–47) will assist in the combination of MI and PI approaches.

## Summary

In this narrative review, we have comprehensively summarized the methodology to explore optimum material compositions using an MI approach. The MI approach promises to accelerate dental material research and contribute to multidisciplinary research in dentistry.

## References

[1] Novikov I Kovalyova O Shapeev A Hodapp M. AI-accelerated materials informatics method for the discovery of ductile alloys. J Mater Res. (2022) 37:3491–504. 10.1557/s43578-022-00783-z

[2] Tamura R Watanabe M Mamiya H Washio K Yano M Danno K Materials informatics approach to understand aluminum alloys. Sci Technol Adv Mater. (2020) 21(1):540–51. 10.1080/14686996.2020.1791676 32939178 PMC7476514

[3] Rickman JM Chan HM Harmer MP Smeltzer JA Marvel CJ Roy A Materials informatics for the screening of multi-principal elements and high-entropy alloys. Nat Commun. (2019) 10(1):2618. 10.1038/s41467-019-10533-1 31197134 PMC6565683

[4] Hara K Yamada S Kurotani A Chikayama E Kikuchi J. Materials informatics approach using domain modelling for exploring structure-property relationships of polymers. Sci Rep-Uk. (2022) 12(1):10558. 10.1038/s41598-022-14394-5 PMC9217937 35732681

[5] Venkatraman V Alsberg BK. Designing high-refractive index polymers using materials informatics. Polymers (Basel). (2018) 10(1):103. 10.3390/polym10010103 30966141 PMC6415069

[6] Nakayama M. Materials informatics for discovery of ion conductive ceramics for batteries. J Ceram Soc Jpn. (2021) 129(6):286–91. 10.2109/jcersj2.21030

[7] Tehrani AM Oliynyk AO Parry M Rizvi Z Couper S Lin F Machine learning directed search for ultraincompressible, superhard materials. J Am Chem Soc. (2018) 140(31):9844–53. 10.1021/jacs.8b02717 30010335

[8] Kim SY Han S Lee S Kang JH Yoon S Park W Discovery of high-performing metal-organic frameworks for on-board methane storage and delivery via LNG-ANG coupling: high-throughput screening, machine learning, and experimental validation. Adv Sci. (2022) 9(21):2201559. 10.1002/advs.202201559 PMC9313482 35524582

[9] Wahl CB Aykol M Swisher JH Montoya JH Suram SK Mirkin CA. Machine learning-accelerated design and synthesis of polyelemental heterostructures. Sci Adv. (2021) 7(52):eabj5505. 10.1126/sciadv.abj5505 34936439 PMC8694626

[10] Rodgers JR. Materials informatics: knowledge acquisition for materials design. Abstr Pap Am Chem Soc. (2003) 226:U302–3.

[11] Senderowitz H Tropsha A. Materials informatics. J Chem Inf Model. (2018) 58(7):1313–4. 10.1021/acs.jcim.8b00016 29638126

[12] National Science and Technology Council (U.S.). Materials genome initiative for global competitiveness. Washington, DC: Executive Office of the President, National Science and Technology Council (2011). Available from: http://purl.fdlp.gov/GPO/gpo9333

[13] LeCun Y Bengio Y Hinton G. Deep learning. Nature. (2015) 521(7553):436–44. 10.1038/nature14539 26017442

[14] Birney E. The making of ENCODE: lessons for big-data projects. Nature. (2012) 489(7414):49–51. 10.1038/489049a 22955613

[15] Gerstein M. Genomics: ENCODE leads the way on big data. Nature. (2012) 489(7415):208. 10.1038/489208b 22972285

[16] Corbella S Srinivas S Cabitza F. Applications of deep learning in dentistry. Oral Surg Oral Med Oral Pathol Oral Radiol. (2021) 132(2):225–38. 10.1016/j.oooo.2020.11.003 33303419

[17] Wang J Wang Y Chen Y. Inverse design of materials by machine learning. Materials (Basel). (2022) 15(5):1811. 10.3390/ma15051811 35269043 PMC8911677

[18] Peterson GGC Brgoch J. Materials discovery through machine learning formation energy. J Phys Energy. (2021) 3(2):022002. 10.1088/2515-7655/abe425

[19] Li H Sakai T Tanaka A Ogura M Lee C Yamaguchi S Interpretable AI explores effective components of CAD/CAM resin composites. J Dent Res. (2022) 101(11):1363–71. 10.1177/00220345221089251 35426349

[20] Wang AYT Murdock RJ Kauwe SK Oliynyk AO Gurlo A Brgoch J Machine learning for materials scientists: an introductory guide toward best practices. Chem Mater. (2020) 32(12):4954–65. 10.1021/acs.chemmater.0c01907

[21] Ferracane JL. Resin composite–state of the art. Dent Mater. (2011) 27(1):29–38. 10.1016/j.dental.2010.10.020 21093034

[22] Ilie N Hilton TJ Heintze SD Hickel R Watts DC Silikas N Academy of dental materials guidance-resin composites: part I-mechanical properties. Dent Mater. (2017) 33(8):880–94. 10.1016/j.dental.2017.04.013 28577893

[23] ISO4049:2019. Dentistry - polymer-based restorative materials. Geneva, Switzerland: International Organization for Standardization (2019).

[24] Okada K Kameya T Ishino H Hayakawa T. A novel technique for preparing dental CAD/CAM composite resin blocks using the filler press and monomer infiltration method. Dent Mater J. (2014) 33(2):203–9. 10.4012/dmj.2013-329 24583649

[25] Yamaguchi S Kani R Kawakami K Tsuji M Inoue S Lee C Fatigue behavior and crack initiation of CAD/CAM resin composite molar crowns. Dent Mater. (2018) 34(10):1578–84. 10.1016/j.dental.2018.07.002 30049596

[26] Ruse ND Sadoun MJ. Resin-composite blocks for dental CAD/CAM applications. J Dent Res. (2014) 93(12):1232–4. 10.1177/0022034514553976 25344335 PMC4462808

[27] Lee C Yamaguchi S Imazato S. Quantitative evaluation of the degradation amount of the silane coupling layer of computer-aided design/computer-aided manufacturing resin composites by water absorption. J Prosthodont Res. (2023) 67(1):55–61. 10.2186/jpr.JPR_D_21_00236 34980788

[28] Li RW Chow TW Matinlinna JP. Ceramic dental biomaterials and CAD/CAM technology: state of the art. J Prosthodont Res. (2014) 58(4):208–16. 10.1016/j.jpor.2014.07.003 25172234

[29] Makhija SK Lawson NC Gilbert GH Litaker MS McClelland JA Louis DR Dentist material selection for single-unit crowns: findings from the national dental practice-based research network. J Dent. (2016) 55:40–7. 10.1016/j.jdent.2016.09.010 27693778 PMC5125852

[30] Garoushi S Sailynoja E Vallittu PK Lassila L. Fracture-behavior of CAD/CAM ceramic crowns before and after cyclic fatigue aging. Int J Prosthodont. (2021). 10.11607/ijp.7207. [Epub ahead of print] 33625389

[31] ISO6872:2015. Dentistry - ceramic materials. Geneva, Switzerland: International Organization for Standardization (2015).

[32] Wang F Yu T Chen J. Biaxial flexural strength and translucent characteristics of dental lithium disilicate glass ceramics with different translucencies. J Prosthodont Res. (2020) 64(1):71–7. 10.1016/j.jpor.2019.04.007 31088735

[33] Souza TJS Freitas ADS Ferreira D Maia LC Rabello TB. Does the use of preheated restorative resin composite as a luting agent influence the adaptation of fixed dental prostheses? A systematic review. J Prosthet Dent. (2022). 10.1016/j.prosdent.2022.02.008. [Epub ahead of print] 35300849

[34] Hill EE. Dental cements for definitive luting: a review and practical clinical considerations. Dent Clin North Am. (2007) 51(3):643–58, vi.. 10.1016/j.cden.2007.04.002 17586148

[35] Leung GK Wong AW Chu CH Yu OY. Update on dental luting materials. Dent J (Basel). (2022) 10(11):208. 10.3390/dj10110208 36354653 PMC9689175

[36] Sakaguchi RL Powers JM. Craig’s restorative dental materials. St. Louis, MO: Elsevier/Mosby (2012).

[37] Kawashima M Yamaguchi S Mine A Li H Imazato S. Novel testing method to evaluate the mechanical strength of self-adhesive resin cements with reflection of cement thickness. Dent Mater J. (2021) 40(5):1235–42. 10.4012/dmj.2020-456 34078779

[38] Kawashima M Mine A Yamaguchi S Imazato S. Development of novel measurement method for consistency of resin cements. Dent Mater J. (2021) 40(4):1063–7. 10.4012/dmj.2020-396 33883356

[39] Géron Al. Hands-on machine learning with Scikit-learn, Keras, and TensorFlow: Concepts, tools, and techniques to build intelligent systems. 2nd ed. Sebastopol: O’Reilly Media (2019). 29 p.

[40] Shields BJ Stevens J Li J Parasram M Damani F Alvarado JIM Bayesian Reaction optimization as a tool for chemical synthesis. Nature. (2021) 590(7844):89–96. 10.1038/s41586-021-03213-y 33536653

[41] Vaucher AC Zipoli F Geluykens J Nair VH Schwaller P Laino T. Automated extraction of chemical synthesis actions from experimental procedures. Nat Commun. (2020) 11(1):3601. 10.1038/s41467-020-17266-6 32681088 PMC7367864

[42] Kim E Jensen Z van Grootel A Huang K Staib M Mysore S Inorganic materials synthesis planning with literature-trained neural networks. J Chem Inf Model. (2020) 60(3):1194–201. 10.1021/acs.jcim.9b00995 31909619

[43] Coley CW Thomas DA 3rd Lummiss JAM Jaworski JN Breen CP Schultz V A robotic platform for flow synthesis of organic compounds informed by AI planning. Science. (2019) 365(6453):eaax1566. 10.1126/science.aax1566 31395756

[44] Burger B Maffettone PM Gusev VV Aitchison CM Bai Y Wang X A mobile robotic chemist. Nature. (2020) 583(7815):237–41. 10.1038/s41586-020-2442-2 32641813

[45] Granda JM Donina L Dragone V Long DL Cronin L. Controlling an organic synthesis robot with machine learning to search for new reactivity. Nature. (2018) 559(7714):377–81. 10.1038/s41586-018-0307-8 30022133 PMC6223543

[46] Li Z Najeeb MA Alves L Sherman AZ Shekar V Parrilla PC Robot-accelerated perovskite investigation and discovery. Chem Mater. (2020) 32(13):5650–63. 10.1021/acs.chemmater.0c01153

[47] Roch LM Hase F Kreisbeck C Tamayo-Mendoza T Yunker LPE Hein JE ChemOS: orchestrating autonomous experimentation. Sci Robot. (2018) 3(19):eaat5559. 10.1126/scirobotics.aat5559 33141686

